# Identification of Potential miRNA-mRNA Regulatory Network Contributing to Parkinson's Disease

**DOI:** 10.1155/2022/2877728

**Published:** 2022-09-05

**Authors:** Xi Yin, Miao Wang, Wei Wang, Tong Chen, Ge Song, Yixuan Niu, Ziying Jiang, Zhongbao Gao, Zhenfu Wang

**Affiliations:** ^1^Department of Neurology, The Second Medical Center and National Clinical Research Center for Geriatric Diseases, Chinese PLA General Hospital, Beijing, China; ^2^Department of Geriatrics, The Second Medical Center and National Clinical Research Center for Geriatric Diseases, Chinese PLA General Hospital, Beijing, China; ^3^Medical School of Chinese PLA, Beijing, China

## Abstract

Parkinson's disease (PD) is a common neurodegenerative disease, and the mechanism underlying PD pathogenesis is not completely understood. Increasing evidence indicates that microRNAs (miRNAs) play a critical regulatory role in the pathogenesis of PD. This study aimed to explore the miRNA-mRNA regulatory network for PD. The differentially expressed miRNAs (DEmis) and genes (DEGs) between PD patients and healthy donors were screened from the miRNA dataset GSE16658 and mRNA dataset GSE100054 downloaded from the Gene Expression Omnibus (GEO) database. Target genes of the DEmis were selected when they were predicted by three or four online databases and overlapped with DEGs from GSE100054. Gene Ontology (GO) and Kyoto Encyclopedia of Genes and Genomes (KEGG) pathway enrichment analysis were then conducted by Database for Annotation, Visualization and Integrated Discovery (DAVID) and Metascape analytic tools. The correlation between the screened genes and PD was evaluated with the online tool Comparative Toxicogenomics Database (CTD), and protein-protein interaction (PPI) networks were built by the STRING platform. We further investigated the expression of genes in the miRNA-mRNA regulatory network in blood samples collected from PD patients and healthy donors via qRT-PCR. We identified 1505 upregulated and 1302 downregulated DEGs, and 77 upregulated and 112 downregulated DEmis were preliminarily screened from the GEO database. Further functional enrichment analysis identified 10 PD-related hub genes, including *RAC1, IRS2, LEPR, PPARGC1A, CAMKK2, RAB10, RAB13, RAB27B, RAB11A*, and *JAK2*, which were mainly involved in Rab protein signaling transduction, AMPK signaling pathway, and signaling by Leptin. A miRNA-mRNA regulatory network was then constructed with 10 hub genes, and their interacting miRNAs overlapped with DEmis, including miR-30e-5p, miR-142-3p, miR-101-3p, miR-32-3p, miR-508-5p, miR-642a-5p, miR-19a-3p, and miR-21-5p. Analysis of clinical samples verified significant upregulation of *LEPR* and downregulation of miR-101-3p and miR-30e-5p in PD patients as compared with healthy donors. Thus, the miRNA-mRNA regulatory network was initially constructed and has the potential to provide novel insights into the pathogenesis and treatment of PD.

## 1. Introduction

Parkinson's disease (PD), the second most common neurodegenerative disease worldwide, is characterized by progressive degeneration of dopaminergic (DA) neurons in substantia nigra pars compacta (SNpc) [1, 2]. It is estimated that about two out of 1000 people of all ages will eventually suffer from this disease [3, 4]. The pathogenesis of PD involves a complicated malfunction of multiple systems, manifested by motor dysfunctions, as well as other nonmotor symptoms [3]. The characteristic motor symptoms of PD include tremors (in the hands, arms, legs, jaw, or head), rigidity (of the limbs and trunk), bradykinesia (of movement), and postural instability [3, 5, 6]. PD is a chronic and progressive disease, suggesting that it persists and worsens over time [4, 7]. Heredity seems to play a role in the pathogenesis of PD, because genetic factors, including heterozygous *GBA* mutations, a-synuclein variants, and tau variants [3, 8, 9], occur in as many as 5% to 10% of PD patients [10]. Environmental factors, such as smoking and alcohol, are also involved in the pathogenesis of PD [3]. Nevertheless, the precise mechanism underlying PD is not fully understood. Recently, epigenetic regulation, such as microRNAs (miRNAs), has emerged as a promising pathological factor of PD [11, 12].

miRNA—short, single-stranded RNA consisting of 20–25 nucleotides—belongs to the noncoding RNA family, which does not translate any proteins [13]. miRNA interacts with the 3′ untranslated regions (3′-UTR) of the target mRNAs, disrupting the translation or accelerating mRNA degradation [14]. Over the past decades, it has been indicated that miRNAs participate in the pathogenesis of various diseases through regulating multiple biological processes, such as cell proliferation, differentiation, and apoptosis [15]. Recent studies have indicated the involvement of miRNAs in PD pathogenesis [12]. For example, miR-124, which regulates NF-*κ*B, STAT3, AMPK, and ERK signaling and subsequent cell apoptosis, autophagy, oxidative damage, and neuroinflammation, was reported to be a potential diagnostic and therapeutic biomarker of PD [16]. Goh et al. implicated several miRNAs, including miR-30, let-7, and miR-485, to be associated with PD pathogenesis [12]. This study highlighted the promising therapeutic role of miRNA in the treatment of PD.

Within the past decade, the development of bioinformatics analyses has not only allowed for the assessment of genetic and epigenetic regulatory pathways associated with diseases but has also offered evidence for strategies aimed at the efficacious diagnosis and treatment of diseases [17, 18]. In the current study, we adopted integrated bioinformatics methods to analyze miRNA and mRNA datasets collected from the Gene Expression Omnibus (GEO) database. From this analysis, we constructed a potential miRNA-mRNA regulatory network associated with PD pathogenesis. We verified the role of this miRNA-mRNA regulatory network in PD by assessing the expression of this pathway in blood samples collected from PD patients and healthy donors via quantitative real-time PCR (qRT-PCR). Our work offers new insights into the pathogenic mechanisms and treatment of PD.

## 2. Materials and Methods

### 2.1. Clinical Samples

Peripheral blood samples were collected from 23 PD patients and 26 healthy donors in heparin-covered tubes, following strict protocol and precautions. All PD patients were diagnosed according to the Movement Disorder Society diagnostic criteria proposed in 2015[19], with a Hoehn and Yahr stage of 2 to 4. All of the healthy donors were age- and gender-matched and were free of diseases, such as dementia, rheumatism, and tumors. All experimental procedures were conducted following the guidelines approved by the Ethics Boards of Chinese PLA General Hospital, Beijing, China. All PD patients and healthy donors provided written informed consent. The information of donors is listed in Supplementary Table 1.

### 2.2. PBMCs Isolation

PBMCs (peripheral blood mononuclear cells) were isolated by Ficoll-Paque PLUS (Haoyang tech, Tianjin, China) centrifugation of heparinized blood. Red blood cells were completely segregated in this isolation process. The time required for isolation of PBMCs was 1-2 hours for the subjects, and samples were frozen at −80°C for qRT-PCR analysis.

### 2.3. qRT-PCR

Total RNA was extracted from isolated PBMCs by the TRIzol Reagent (Invitrogen, Carlsbad, CA, USA) following the manufacturer's protocol. qRT-PCR was conducted via Fast SYBR® Green Master Mix Bulk Pack (Invitrogen, Carlsbad, CA, USA) according to the manufacturer's instructions. Small endogenous nuclear *U6* snRNA and *GAPDH* were used as an internal control for the normalization of miRNAs and mRNAs, respectively. Gene expression levels were calculated by the (2^−ΔΔCt^) method. The sense and antisense primers used in this study are as follows:

  GAPDH:sense,5′-GGAGCGAGATCCCTCCAAAAT-3′,antisense,5′-GGCTGTTGTCATACTTCTCATGG-3′
  U6: sense, 5′-CTCGCTTCGGCAGCACA-3′, antisense, 5′- AACGCTTCACGAATTTGCGT-3′
  LEPR: sense, 5′-TGGGATTAGGTGGGATTT-3′, antisense, 5′-CCGCTCCTACCAATCTAA-3′
  IRS2:sense,5′‐TTGACTTCTTGTCCCACCACTTG‐3′,antisense,5′‐GCTGAG CGTCTTCTTTTAATGATACT‐3′
  JAK2: sense, 5′-TCTGGGGAGTATGTTGCAGAA-3′, antisense, 5′-AGACATGGTTGGGTGGATACC-3′
  miR-30e-5p: sense, 5′-GGGTGTAAACATCCTTGAC-3′, antisense, 5′-TGCGTGTCGTGGAGTC-3′
  miR-101-3p:sense,5′-GCCGCCACCATGGTGAGCAAGG-3′,antisense,5′-AATTGAAAAAAGTGATTTAATTT-3′

### 2.4. Microarray Data

The miRNA-expressing dataset GSE16658 and mRNA-expressing dataset GSE100054 were downloaded from the online GEO database (https://www.ncbi.nlm.nih.gov/geo/). All samples were PBMCs collected from PD patients and healthy donors. The GSE16658 dataset consisted of 32 samples, among which 19 were from PD patients and 13 were from healthy donors. The GSE100054 dataset contained 19 samples, including 10 from PD patients and 9 from healthy donors. The GSE16658 and GSE100054 datasets were detected by the Illumina Human v2 MicroRNA Expression BeadChip platform and Illumina HumanHT-12 V3.0 Expression BeadChip platform (Illumina, San Diego, CA, USA), respectively.

### 2.5. Identification of Differentially Expressed Genes (DEGs) and miRNAs (DEmis)

In order to obtain differentially expressed genes (DEGs) and miRNAs (DEmis) from PD patients and healthy control, the raw data from the GSE16658 and GSE100054 datasets were analyzed by GEO2R, a GEO-provided analytic tool. The |log2FC| ≥ 1 and *p* < 0.05 were introduced as cut-off criteria. The DEGs and DEmis were visualized by volcano plots.

### 2.6. Prediction of miRNA Targets

Targets of the selected upregulated and downregulated DEmis from GSE16658 were predicted by four online databases, including miRDB (https://mirdb.org/miRDB/index.html), TargetScan (https://targetscan.org/vert_72/), miRWalk (https://mirwalk.umm.uni-heidelberg.de/), and miRTarBase databases (https://mirtarbase.mbc.nctu.edu.tw). The targets, which were simultaneously predicted by at least three databases and overlapped with the DEGs from GSE100054, were selected for further study.

### 2.7. Construction of the miRNA-mRNA Regulatory Network

The miRNA-mRNA regulatory network was established and visualized by Cytoscape version 3.7.2 software (https://www.cytoscape.org/) [20] using the selected miRNAs and overlapping target genes.

### 2.8. Functional Enrichment Analysis

We conducted Gene Ontology (GO) analysis (biological process, molecular function, and cellular component) and Kyoto Encyclopedia of Genes and Genomes (KEGG) analysis to identify biological functions and pathways enriched from the predicted target genes of the DEmis. The functional enrichment analysis was conducted by using the online bioinformatics tool, the Database for Annotation, Visualization and Integrated Discovery (DAVID, https://david.ncifcrf.gov/). The terms with adjusted *p* values <0.05 were selected.

The enriched functional terms were also determined by the Metascape online analytic tool (https://metascape.org/gp/index.html#/main/step1) based on several databases, such as GO, KEGG, Reactome, and CORUM. The top 20 enriched terms were visualized as an enrichment bar graph.

### 2.9. Comparative Toxicogenomics Database (CTD)

The correlation between the screened genes and PD was evaluated by the online tool CTD (https://ctdbase.org/), scored, and shown as bar graphs.

### 2.10. Protein-Protein Interaction (PPI) Network

The protein-protein interaction (PPI) network was built by the online platform Search Tool for the Retrieval of Interacting Genes (STRING, https://string-db.org/) (version 11.0) [21] to analyze the connection of target genes of the DEmis. The interacted pairs with a confidence score ≥0.4 were preserved and visualized by Cytoscape software [20].

### 2.11. Statistical Analysis

The data were presented as the mean ± standard deviation (SD). Student's *t*-test and one-way ANOVA were used to assess the difference between two groups and multiple groups, respectively. *p* < 0.05 was considered as the statistical threshold.

## 3. Results

### 3.1. Identification of DEGs and DEmis in PD

**Figure 1:**
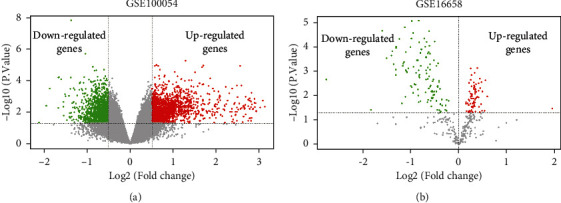
Identification of DEGs and DEmis between PD patients and healthy controls. (a) Volcano plots of 1505 upregulated DEGs and 1302 downregulated DEGs in PD patients (a) and 77 upregulated DEmis and 112 downregulated DEmis were identified in PD patients (b) compared with the control groups. *p* < 0.05 is taken as a threshold. Blue dots represent upregulated genes or miRNAs, red dots indicate upregulated ones, and grey dots represent no significance. The abscissa axis represents log2 |fold change|, and the vertical axis represents −log10 |(*p*) value|.

In order to determine critical genes and miRNAs involved in the progression of PD, we analyzed the PBMC samples of the mRNA dataset GSE100054 and miRNA dataset GSE16658. We obtained 1505 significantly upregulated DEGs and 1302 downregulated DEGs in PD patients as compared with healthy donors (Figure 1(a)). In addition, 77 upregulated DEmis and 112 downregulated DEmis were identified in PD patients (Figure 1(b)), among which the top ten were selected for further analysis (Table 1).

### 3.2. Target Prediction of DEmis

**Figure 2:**
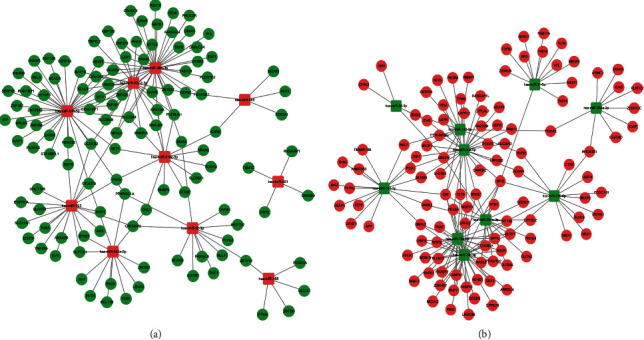
miRNA target prediction and selection. The target prediction of each miRNA was conducted using miRDB, TargetScan, miRWalk, and miRTarBase databases. (a) The target genes of upregulated DEmis commonly predicted by 3 and 4 databases and simultaneously overlapped with the downregulated DEGs from the GSE100054 dataset were selected to construct the regulatory network. (b) The regulatory network of downregulated DEmis and their target genes selected from overlaps between upregulated DEGs from GSE100054 dataset and predicted targets by 3 and 4 databases.

After identifying the top 10 DEmis, we tried to determine their target genes. Four online tools, namely, the miRDB, TargetScan, miRWalk, and miRTarBase databases, were utilized to predict the potential targets. The target genes of the upregulated DEmis predicted by at least three databases were overlapped with the downregulated DEGs from the GSE100054 dataset, and the shared genes between these overlaps were further selected to build a potential regulatory network (Figure 2(a)). A similar screening was performed on the downregulated DEmis and upregulated DEGs (Figure 2(b)). This analysis provided a preliminary screening for the prognostic miRNA-mRNA regulatory network of PD.

### 3.3. Functional Enrichment Analysis of the DEGs in PD

**Figure 3:**
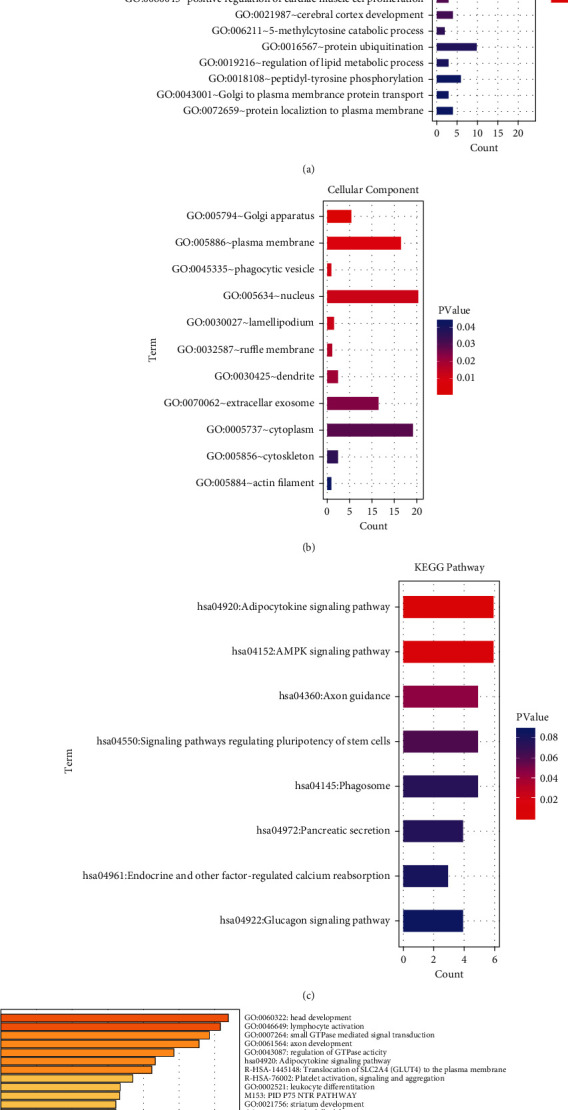
Functional enrichment analysis via DAVID and Metascape database. GO analysis was performed on the target genes of the DEmis, and the most significantly enriched biological process terms (a) and the molecular function terms (b) were presented as bar graphs. KEGG signaling pathways enriched from the target genes of the DEmis (c). The enriched functional terms enriched from target genes of the DEmis were determined by Metascape online analytics tool based on several databases such as GO, KEGG, Reactome, and CORUM. The top 20 enriched terms were visualized as an enrichment bar graph (d).

In order to optimize the critical genes and miRNAs associated with the development of PD, we conducted functional enrichment analysis to screen the significant biological processes and signaling pathways and selected the genes involved in these processes. We first introduced two bioinformatics tools, the DAVID and Metascape databases, to conduct the functional enrichment analysis on the predicted targets of DEmis (mentioned above in Figure 2). As shown in Figure 3, the results from the DAVID database revealed the representative GO terms, which included biological processes (Figure 3(a)), cellular component (Figure 3(b)), and KEGG signaling pathways (Figure 3(c)). Another enrichment analysis performed through the Metascape website revealed the significant functional processes based on the GO, KEGG, Reactome, and CORUM databases, and the top 20 terms are shown in the bar graph (Figure 3(d)).

Subsequently, we optimized the PD-related terms from the enrichment outcome of DAVID and Metascape analysis by searching each term together with the term “Parkinson” on the PubMed website. As listed in Table 2, four terms were identified for further analysis, including the Rab GTPase binding, AMPK signaling pathway, Rab protein signaling transduction, and signaling by Leptin.

### 3.4. Selection of Hub Genes Related to PD Pathogenesis

**Figure 4:**
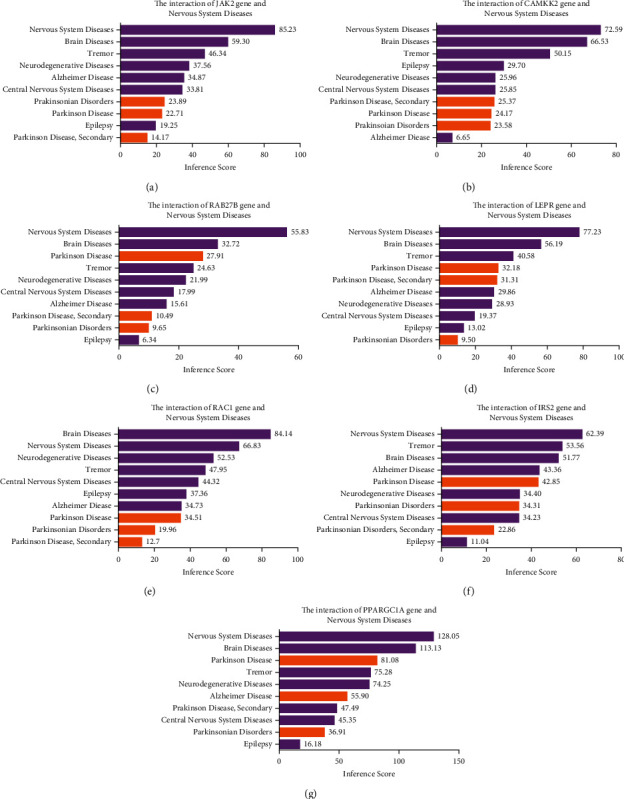
Parkinson's disease score determined by CTD. The correlation between the genesis of PD and *JAK2* (a), *CAMKK2* (b), *RAB27B* (c), *LEPR* (d), *RAC1* (e), *IRS2* (f), and *PPARGC1A* (g) was evaluated by the online tool Comparative Toxicogenomics Database (CTD), scored, and shown as bar graphs.

Among the genes involved in the selected four functional terms, 10 were previously reported in PD studies, including *RAC1, IRS2, LEPR, PPARGC1A, CAMKK2, RAB10, RAB13, RAB27B, RAB11A*, and *JAK2*. To confirm their functional roles in PD, we employed the CTD database to confirm their correlation with nervous system diseases via evaluating the disease score. The genes with a PD score >20 are identified as significantly correlated genes and are marked as orange bars in Figure 4. Among these 10 genes, *JAK2* (Figure 4(a)), *CAMKK2* (Figure 4(b)), *RAB27B* (Figure 4(c)), *LEPR* (Figure 4(d)), *RAC1* (Figure 4(e)), *IRS2* (Figure 4(f)), and *PPARGC1A* (Figure 4(g)) scored 22.71, 24.17, 27.91, 32.18, 34.51, 42.85, and 81.08, respectively, and were considered to be crucial PD-related genes. *RAB10, RAB13*, and *RAB11A* were excluded, due to their low scores (Supplementary Figure 1).

**Figure 5:**
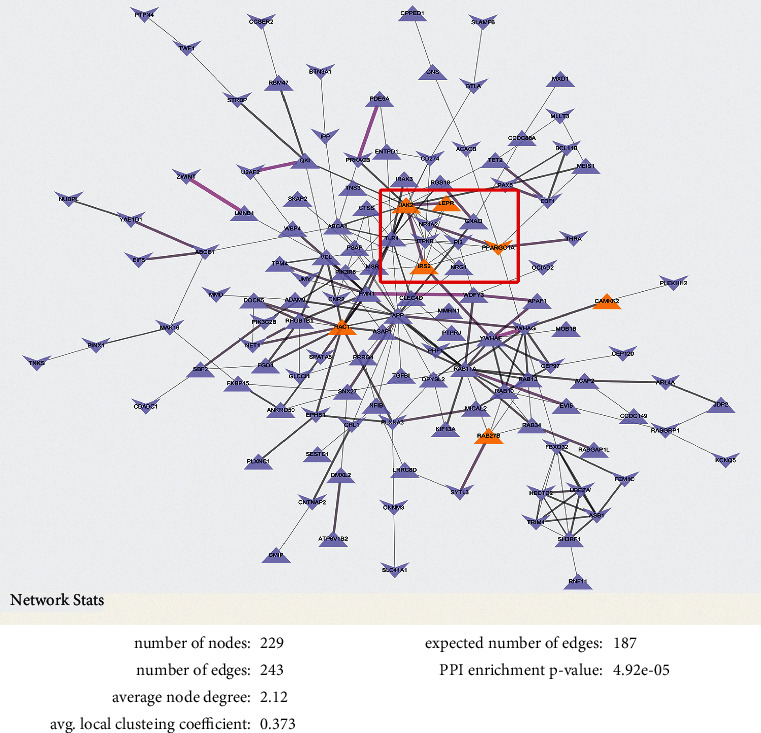
Construction of PPI network. The protein-protein interaction (PPI) network was constructed via Search Tool for the Retrieval of Interacting Genes (STRING) and visualized by Cytoscape software. A confidence score ≥0.4 was regarded as the criterion.

To confirm the hub genes related to PD, we also established a PPI network based on the target genes of the DEmis. Intriguingly, the PPI network showed that four of the seven CTD-selected genes, *IRS2, LEPR, JAK2,* and *PPARGC1A*, were closely correlated, suggesting their potential regulatory functions in PD development (Figure 5).

### 3.5. Determination and Validation of the Critical miRNA-mRNA Regulatory Network in Clinical PD Samples

**Figure 6:**
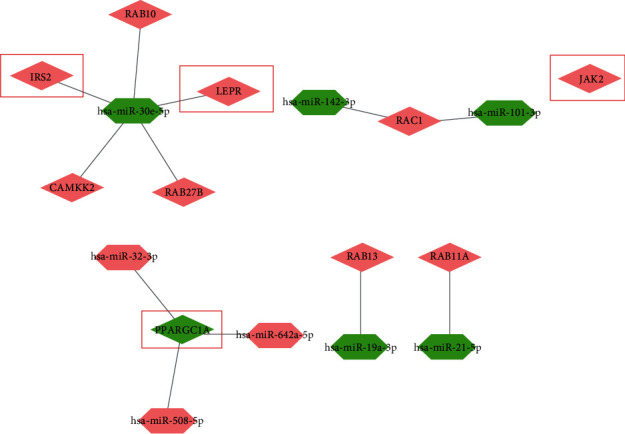
Optimized miRNA-mRNA regulatory network. The regulatory network consists of 10 optimized genes, *JAK2, CAMKK2, RAB27B, LEPR, RAC1, IRS2*, and *PPARGC1A*, and their corresponding miRNAs, miR-30e-5p, miR-142-3p, miR-101-3p, miR-32-3p, miR-508-5p, miR-642a-5p, miR-19a-3p, and miR-21-5p.

**Figure 7:**
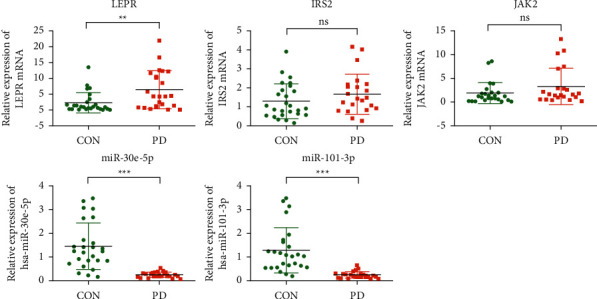
Evaluation of the miRNA-mRNA regulatory network in clinical PD samples. Real-time PCR was performed to examine the RNA levels of miR-30e-5p, miR-101-3p, *JAK2, LEPR*, and *IRS2* in the peripheral blood samples of 23 PD patients and 26 healthy donors. ^*∗*^*p* < 0.05, ^*∗∗*^*p* < 0.01, and ^*∗∗∗*^*p* < 0.001 vs. control (CON).

Based on the abovementioned screening and selection, we finally constructed a simplified miRNA-mRNA regulatory network consisting of 10 PD-related genes and their interacting miRNAs selected from the DEmis (Figure 6), including miR-30e-5p, miR-142-3p, miR-101-3p, miR-32-3p, miR-508-5p, miR-642a-5p, miR-19a-3p, and miR-21-5p. We next collected blood samples from PD patients and healthy donors to examine the expression of the hub genes and their interacting miRNAs. Among the hub genes and their interacting miRNAs, the mRNA level of *IRS2, LEPR,* and *JAK2* was upregulated, while the expression of miR-101-3p and miR-30e-5p was downregulated in PD patients. The changes in *LEPR*, miR-30e-5p, and miR-101-3p were statistically different (Figure 7).

## 4. Discussion

PD is a common age-related neurodegenerative disorder characterized by the progressive degeneration of the dopaminergic neurons in substantia nigra pars compacta (SNpc) [3, 22]. While the mechanism underlying PD pathogenesis is still poorly understood [1, 4], it is believed that PD results from a complicated interplay of genetic and environmental factors affecting numerous fundamental cellular processes [10]. Moreover, epigenetic factors, such as miRNAs, have been shown to be important biological molecules involved in the pathogenesis of PD [11, 12]. In this study, we used miRNA and mRNA datasets from the GEO database, conducted multiple bioinformatics analyses to construct a potential miRNA-mRNA regulatory network, identified several signaling pathways and hub genes that are associated with PD development, and further verified these hub genes via qRT-PCR in the blood samples of PD patients and healthy donors. We found an upregulation of *LEPR* and downregulation of miR-30e-5p/miR-101-3p in PD patients as compared to healthy donors.

miRNAs have emerged as pivotal molecules involved in PD pathogenesis. A previous study suggested that miR-425 deficiency triggers necroptosis of dopaminergic neurons, and targeting miR-425 in a murine model of PD restored dysfunctional dopaminergic neurodegeneration and ameliorated behavioral deficits [23]. In addition, miR-27a and miR-27b were thought to regulate autophagic clearance of damaged mitochondria and *PINK1* gene expression, which is the most common cause of autosomal recessive PD [24]. Moreover, the miRNAs analyzed in our study, including miR-30e, miR-21, miR-101, miR-19a, and miR-142, which were downregulated, were previously presented as regulators of PD [25, 26]. The miR-30 family has been investigated in multiple studies and seems to play a critical role during PD [12, 27, 28]. Bioinformatics analysis on various patient samples of PD identified the miR-30 family as potential upstream regulators of progression rate-related biomarkers of PD [29]. Moreover, miR-30e was reported to attenuate the levels of inflammatory cytokines, such as TNF-*α*, COX-2, and iNOS, and ameliorate neuroinflammation in a MPTP model of PD through directly targeting the *NLRP3* inflammasome [30]. miR-101-3p was previously reported to mediate lncRNA Mirt2-suppressed inflammation [31]. Our study showed a reduced level of miR-30e-5p and miR-101-3p in the PBMCs of PD patients, supporting its negative regulatory role during PD, which was consistent with previous studies. Based on these studies and including our own, the existence of a miRNA-mRNA regulatory network in PD pathogenesis is believed.

Traditionally, miRNAs function by regulating target genes in a posttranscriptional manner and play critical roles in various biological processes. We further listed the members of these enriched functional terms, predicted their potential interacting mRNAs, obtained the overlaps with the DEGs, and then constructed a miRNA-mRNA regulatory network. It is worth noting that four of the genes among the regulatory network, *IRS2, LEPR, JAK2,* and *PPARGC1A,* were identified as the hub genes, suggesting their critical roles during PD progression. The three upregulated genes, *IRS2, LEPR,* and *JAK2,* participate in leptin-mediated metabolism and inflammatory regulation in PD, which is consistent with their roles in fatty acid transport and gluconeogenesis, further highlighting the important role of leptin signaling in PD progression [32, 33]. Moreover, we identified *LEPR* as a significantly elevated gene in PD patients as compared with the healthy controls. Protein leptin encoded by the gene *LEPR* serves as a regulator of energy homeostasis and feeding behavior [34] and exhibits neurotrophic actions during the perinatal development of the central nervous system during and into adulthood [35]. Ho et al. showed that leptin preserved cell survival in neuronal SH-SY5Y cells against MPP + toxicity (Parkinson's disease model) by maintaining ATP levels and mitochondrial membrane potential. The upregulation of the *LEPR* gene might indicate activated feedback protection by the neurons, which will be investigated in future studies with a larger PD cohort. The *PPARGC1A* gene, which encodes the transcriptional coactivator, PGC-1*α*, which has been implicated in the pathogenesis of neurodegenerative disorders and found repressed in PD [36], was downregulated in our study. Targeting PGC-1*α* was proposed as a potential therapeutic method for PD [37]. Interestingly, PGC-1*α* was also reported to regulate the *IRS2* level in hepatic metabolism [38]. In our study, the *PPARGC1A* gene interacted with three different miRNAs, and as such, its role in PD pathogenesis might be more complicated than originally thought. Further study will be conducted to investigate the exact role of the *PPARGC1A* gene in the pathogenesis of PD.

By functional enrichment analysis, we screened out biological signaling pathways closely associated with PD, including the Rab protein signaling transduction, AMPK signaling pathway, and signaling by Leptin. The hub genes, *IRS2, LEPR, JAK2,* and *PPARGC1A,* were mainly involved in AMPK and Leptin signaling pathways. Significant evidence from PD models has supported the participation of AMPK in PD, via regulating cellular metabolism, enhancing autophagy, promoting mitochondrial quality control, suppressing oxidation, and alleviating inflammation [39–41]. The AMPK activation was therefore regarded as a therapeutic target for PD treatment [39]. Moreover, it has been demonstrated that AMPK activation may facilitate clearance of *α*-synuclein, thereby promoting neuronal survival to ameliorate PD [42]. Abnormal leptin signaling was also frequently observed in neurodegeneration diseases [43]. Increasing evidence has presented the role of leptin in regulating metabolic homeostasis during PD [43, 44]. Candia et al. also suggested that leptin played a key role in linking metabolic imbalance and damage to the nervous system [44]. In addition, leptin was also involved in blood pressure changes during orthostatic stress in PD patients [45]. Our research and previous evidence have indicated that the interaction of miRNAs and mRNAs, which build a regulatory network and function in various signaling pathways, participate in the pathogenesis of PD.

As a whole, our study provides a comprehensive study on the role of miRNAs and mRNAs in PD. We identified a potential miRNA-mRNA regulatory network correlated with PD pathogenesis by using bioinformatics tools and verified this network in blood samples collected from PD patients. The results of this study have the potential to provide novel insights into the pathogenesis and potential therapeutic targets of PD.

## 5. Conclusions

In conclusion, based on the bioinformatics tools and blood sample verification, a potential miRNA-mRNA regulatory network correlated with PD was identified in the study. These findings could pave the way to identify new approaches for the treatment of PD.

## 6. Disclosure

An earlier version of the manuscript has been presented as Preprint on https://www.researchsquare.com [46].

## Figures and Tables

**Table 1:** 10 upregulated miRNAs and 10 downregulated miRNAs were screened in GSE16658 dataset.

ID	*p* value	logFC	miRNA ID
10 upregulated miRNAs			
42812	0.03403022	1.948885	hsa-miR-508-5p
42762	0.00241734	0.55537	hsa-miR-665
17836	0.04180705	0.481978	hsa-miR-30b-3p
42785	0.00280149	0.473074	hsa-miR-921
42701	0.02314422	0.463982	hsa-miR-30c-2-3p
28150	0.01187665	0.412631	hsa-miR-765
29575	0.0063856	0.412492	hsa-miR-32-3p
42727	0.01131039	0.386259	hsa-miR-668
42679	0.00076206	0.382502	hsa-miR-642a-5p
13131	0.00741607	0.362588	hsa-miR-518c-5p

10 downregulated miRNAs			
17660	0.00219334	−2.754213	hsa-miR-550a-3p
33596	0.00002234	−1.592887	hsa-miR-126-5p
28191	0.00029506	−1.497504	hsa-miR-30e-5p
31026	0.00023161	−1.398265	hsa-miR-101-3p
10997	0.00015777	−1.383207	hsa-miR-19a-3p
10947	0.00052467	−1.353895	hsa-miR-142-3p
11053	0.00090354	−1.313697	hsa-miR-32-5p
5740	0.00147611	−1.306943	hsa-miR-21-5p
13143	0.00011819	−1.306042	hsa-miR-301a-3p
10998	0.000103	−1.30473	hsa-miR-19b-3p

**Table 2:** Four terms related to PD.

Term	Literature number	Count	*p* value	Genes
GO:0017137∼Rab GTPase binding	109	6	0.026593331	*DMXL2, EVI5, ACAP2, RAC1, RABGAP1L,* and *SYTL3*
hsa04152: AMPK signaling pathway	53	6	0.009607244	*IRS2, LEPR, ACACB, RAB10, PPARGC1A,* and *CAMKK2*
Rab protein signal transduction	27	5	0.000709612	*RAB13, RAB27B, RAB11A, RAB10,* and *RAB34*
Signaling by Leptin	11	3	0.000127852	*JAK2, LEPR,* and *IRS2*

## Data Availability

The datasets used during the current study are available from the corresponding author on reasonable request.

## Supplementary Materials

Supplementary Materials
*Supplementary Figure 1.* Parkinson's disease scores of RAB 10, RAB 13, and RAB 11A are determined by CTD. *Supplementary Table 1.* Clinical information of PD patients and healthy donors involved in the study.
