# Supplementary material for: Identification of Potential miRNA-mRNA Regulatory Network Contributing to Parkinson's Disease
Source: Parkinsons Dis. 2022 Sep 5;2022:2877728. doi: 10.1155/2022/2877728 (PMC9467752; doi:10.1155/2022/2877728)
Supplement: Supplementary Materials — Supplementary Figure 1. Parkinson's disease scores of RAB 10, RAB 13, and RAB 11A are determined by CTD. Supplementary Table 1. Clinical information of PD patients and healthy donors involved in the study. [file 2877728.f1.pdf]

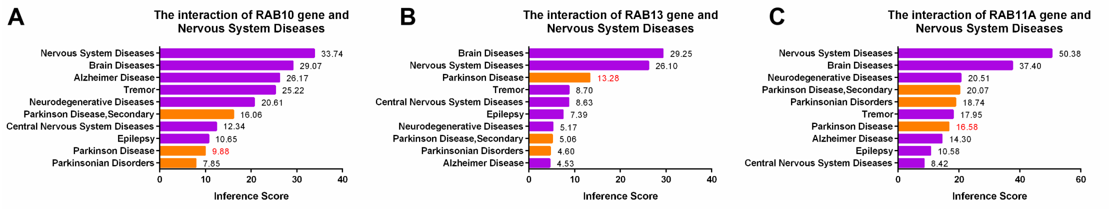

Supplementary Figure 1 Parkinson Disease score determined by CTD. The correlation between the genesis of PD and RAB10 (**A**), RAB13 (**B**) and RAB11A (**C**) was evaluated by the online tool Comparative Toxicogenomics Database (CTD), scored and showed as bar graphs.

Supplementary Table 1. Clinical information.

ID	Sex	Age	Hoehn and Yahr stage
PD-1	Male	89	2
PD-2	Female	94	4
PD-3	Male	68	3
PD-4	Female	93	4
PD-5	Male	78	3
PD-6	Female	74	2
PD-7	Female	67	2
PD-8	Male	69	4
PD-9	Male	71	2
PD-10	Male	68	3
PD-11	Female	75	2
PD-12	Male	57	4
PD-13	Female	59	3
PD-14	Male	84	2
PD-15	Female	72	3
PD-16	Male	68	3
PD-17	Male	69	2
PD-18	Female	61	3
PD-19	Female	69	3

PD-20	Male	64	3
PD-21	Female	66	2
PD-22	Female	58	2
PD-23	Male	74	3
CON-1	Male	69	——
CON-2	Female	86	——
CON-3	Female	62	——
CON-4	Female	62	——
CON-5	Male	90	——
CON-6	Male	82	——
CON-7	Female	81	——
CON-8	Female	95	——
CON-9	Female	75	——
CON-10	Male	61	——
CON-11	Male	62	——
CON-12	Female	62	——
CON-13	Male	63	——
CON-14	Female	68	——
CON-15	Male	60	——
CON-16	Male	61	——

CON-17	Male	61	——
CON-18	Female	64	——
CON-19	Female	66	——
CON-20	Male	66	——
CON-21	Male	67	——
CON-22	Female	67	——
CON-23	Male	67	——
CON-24	Female	69	——
CON-25	Female	59	——
CON-26	Male	64	——
